# Endoscopic excision for internal and mixed hemorrhoids: a retrospective case series of short-term outcomes

**DOI:** 10.3389/fphys.2026.1762846

**Published:** 2026-02-19

**Authors:** Qi Xu, Bingfeng Qiu, Tangzhou Xu, Dandan Zhuang, Junhan Qu

**Affiliations:** Department of Gastroenterology, Zhoushan Hospital, Zhejiang University School of Medicine, Zhoushan, Zhejiang, China

**Keywords:** endoscopic excision, internal hemorrhoids, mixed hemorrhoids, safety, short-term clinical outcomes analysis

## Abstract

**Objective:**

This study aimed to investigate the short-term clinical outcomes and safety of endoscopic excision for the management of internal and mixed hemorrhoids.

**Methods:**

A retrospective analysis was conducted on 20 patients with Grade II to Grade IV internal or mixed hemorrhoids who underwent endoscopic excision at Zhoushan Hospital between January 2024 and December 2024. All patients had complete follow-up data.

**Results:**

At 3 and 6 months after surgery, the treatment effectiveness rate was 100%, and both postoperative satisfaction and acceptance rates were 100%. No severe postoperative complications occurred, and no bleeding or infection was observed. Mild pain developed in three patients, a transient sensation of anal heaviness and distension occurred in one patient, and temporary urinary retention occurred in one patient, which resolved after local hot compress therapy. Postoperative pathological examinations confirmed that the resected anorectal masses demonstrated changes consistent with hemorrhoidal tissue.

**Conclusion:**

Endoscopic excision for internal and mixed hemorrhoids is a safe and effective therapeutic approach. It provides significant symptom relief, yields high postoperative satisfaction and acceptance among patients, and allows for definitive pathological confirmation of the nature of the resected anorectal tissue.

## 1 Introduction

Hemorrhoids are among the most common anorectal conditions encountered in clinical practice and are classified into internal hemorrhoids, external hemorrhoids, and mixed hemorrhoids. Internal hemorrhoids account for the highest proportion of hemorrhoidal diseases (59.86%), with most cases categorized as Grade I to Grade III internal hemorrhoids (99.47%) (Chinese Society of Digestive Endoscopology, 2021). The primary symptoms include bleeding, prolapse, pain, itching, and difficulty with defecation, which substantially affect patients’ daily functions and quality of life.

Conventional management of internal and mixed hemorrhoids typically involves pharmacologic therapy and surgical interventions. With recent advancements in endoscopic techniques, endoscopic ligation and sclerotherapy have been increasingly applied to internal hemorrhoids and have demonstrated favorable outcomes (Jiang et al., 2021). However, for patients with Grade II or Grade III internal hemorrhoids complicated by mucosal prolapse, as well as those with mixed hemorrhoids, the therapeutic effects of ligation and sclerotherapy remain suboptimal (Davis et al., 2018). Given these challenges, the Department of Gastroenterology of Zhoushan Hospital implemented endoscopic excision for internal and mixed hemorrhoids beginning in 2024. The present retrospective evaluation included 20 patients who underwent endoscopic excision in 2024 to assess the safety and effectiveness of this technique and to provide new perspectives and potential strategies for the treatment of internal and mixed hemorrhoids.

## 2 Participants and methods

### 2.1 Research participants

From January 2024 to December 2024, 20 patients with internal or mixed hemorrhoids were admitted and hospitalized at Zhoushan Hospital. The cohort included 11 males and nine females, aged 32–65 years, with a mean age of 49.8 ± 1.3 years. At admission, 12 patients presented with hematochezia, 5 with prolapse, 2 with pain, and 1 with difficulty in defecation. Internal hemorrhoids were graded according to the Goligher classification (Goligher, 1984). Among these patients, three had Grade II internal hemorrhoids (all accompanied by mucosal prolapse), eight had Grade III internal hemorrhoids, and six had Grade IV internal hemorrhoids. Additionally, three patients had mixed hemorrhoids (Table 1).

**TABLE 1:** Basic clinical information of patients with internal or mixed hemorrhoids.

Items	N	Percentage (%)
Total cases	20	-
Sex composition		
Male	11	55.0
Female	9	45.0
Symptoms at admission		
Hematochezia	12	60.0
Prolapse	5	25.0
Pain	2	10.0
Difficulty in defecation	1	5.0
Pathological classification (Goligher grading)		
Grade II internal hemorrhoids	3	15.0
Grade III internal hemorrhoids	8	40.0
Grade IV internal hemorrhoids	6	30.0
Mixed hemorrhoids	3	15.0

### 2.2 General clinical data

Based on the LDRF classification proposed by Linghu et al., the 20 patients with internal or mixed hemorrhoids were categorized accordingly (Table 2) (Linghu et al., 2020).

**TABLE 2:** LDRF classification results for patients with internal or mixed hemorrhoids.

Classification	Clinical features	n	Percentage (%)
RF0	Negative red sign	8	40.0
RF1	Positive red sign, no erosion, thrombus, or active bleeding	9	45.0
RF2	Surface mucosa has erosion, thrombus, and active bleeding	3	15.0
Total		20	100.0

The diameter of the hemorrhoid ranges from 0.8 to 1.5 cm.

### 2.3 Research instruments

The instruments included the Olympus 290 electronic endoscopy system (Japan), injection needles, snares, transparent caps, and water pumps.

### 2.4 Treatment methods

Preoperative preparation: All patients underwent routine blood tests, biochemical tests, and coagulation assessments after admission to evaluate potential surgical contraindications. For patients without contraindications, bowel preparation was performed, followed by routine colonoscopy to exclude other intestinal conditions. Endoscopic polypectomy was conducted if polyps were identified. Endoscopic treatment was subsequently performed for internal and mixed hemorrhoids.

**FIGURE 1:**
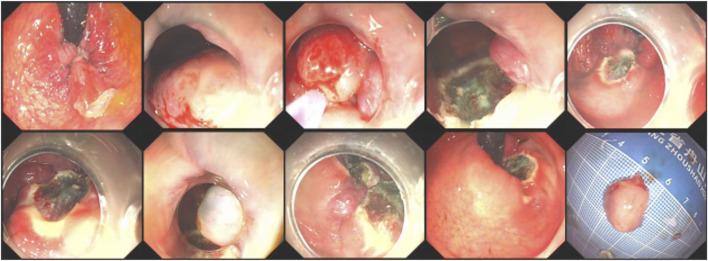
Endoscopic excision procedure for internal and mixed hemorrhoids.

Procedure steps: Patients were placed in the left lateral position. Under endoscopic visualization, the degree, location, and extent of involvement of internal and mixed hemorrhoids were thoroughly assessed. Submucosal injection of a mixed solution containing methylthionine chloride and adrenaline in saline was administered at the base of the hemorrhoids at the 3, 6, 9, and 12 o’clock positions. After mucosal elevation, a snare was used to encircle the venous clusters for high-frequency electrocautery resection. Hemostasis was achieved using hot biopsy forceps, as presented in Figure 1. For patients with prolapse, an assistant is usually asked to insert their hand into the anus before performing the removal. If the hand cannot be inserted, the removal is carried out directly. For cases of mixed hemorrhoids with external hemorrhoid components that require skin excision, after subcutaneous injection of methylene blue normal saline, the ring forceps are used for direct excision (Supplementary Video S1).

Postoperative management: After surgery, patients were allowed to consume liquid and semi-liquid diets. They were instructed to rest in a supine position, avoid prolonged sitting or standing, and refrain from strenuous activities. Erythromycin ointment was applied externally for 72 h to prevent infection. Patients were monitored in the hospital for 24 h following surgery. By 2024, postoperative administration of anti-inflammatory suppositories for enema will be given. Generally, the pain will be significantly relieved within 24 h. If there is no relief, pirexib injection will be administered by intramuscular injection. For patients who had undergone polyp removal and were suspected of malignancy, the decision on whether to proceed with additional surgery would be made based on the pathology report. Of course, for those with suspected polyp canceration that cannot be completely removed by endoscopy, a hemorrhoidectomy would be arranged first, followed by referral to the surgical department for colon tumor resection.

### 2.5 Observation indicators

1. Postoperative complications: These primarily included sensations of anal heaviness and distension, bleeding, infection, pain (evaluated using the Visual Analog Scale (VAS), where one to three points indicate mild pain, 4–6 points indicate moderate pain, and 7–10 points indicate severe pain), urinary retention, and difficulty in defecation.
2. Patient satisfaction and acceptance of surgery.
3. Treatment effectiveness: Treatment effectiveness was defined as complete resolution of symptoms rate plus improvement rate. Complete resolution of symptoms referred to such as hematochezia and prolapse after treatment, with internal or mixed hemorrhoids reduced by at least one grade, hemorrhoidal size decreased by at least 50%, and bleeding improved by at least 50% compared with pretreatment findings. Improvement referred to significant alleviation of the above symptoms after treatment, with internal or mixed hemorrhoids reduced by at least one grade but with less than a 50% reduction in hemorrhoidal size and less than a 50% improvement in bleeding compared with pretreatment findings. Ineffectiveness referred to not meeting the above criteria or experiencing worsening symptoms after treatment.

### 2.6 Statistics analysis

Due to the small sample size and the nature of the research design, no inferential statistical analysis for inter-group comparisons was conducted, nor was a confidence interval calculated.

## 3 Results

### 3.1 Postoperative complications

No severe complications occurred among the 20 patients, and no postoperative bleeding or infection was observed. Mild postoperative pain developed in three patients, a sensation of anal heaviness and distension occurred in one patient, and urinary retention occurred in one patient. Urinary retention resolved promptly after the application of local hot compresses (Table 3).

**TABLE 3:** Postoperative complications following endoscopic excision.

Types of complications	n	Incidence (%)	Management
Severe complications			
Postoperative bleeding	0	0.0	-
Postoperative infection	0	0.0	-
Mild complications			
Mild postoperative pain	3	15.0	Self-relieved
Sensation of anal heaviness and distension	1	5.0	Self-relieved
Urinary retention	1	5.0	Relief after local hot compress
Overall complication occurrence	5	25.0	
No complication	15	75.0	

### 3.2 Treatment effectiveness

At 3 months after surgery, hematochezia, prolapse, and other clinical symptoms had completely resolved in all 20 patients, and all met the criteria for complete resolution of symptoms. At the time of reporting, eight patients had follow-up periods of less than 6 months, and the remaining 12 patients had completed 6 months of follow-up. All patients who completed the 6-month follow-up were considered complete resolution of symptoms. The treatment effectiveness rate at both 3 months and 6 months after surgery was 100%. Continued follow-up to 12 months postoperatively is required for all patients (Table 4).

**TABLE 4:** Postoperative follow up treatment effectiveness after endoscopic excision.

Follow-up time	Number of cases to be followed up	Actual number of follow-up cases	Improvement of clinical symptoms	Number of cured cases	Treatment effectiveness (%)
3 months after surgery	20	20	Symptoms such as hematochezia and prolapse have completely disappeared	20	100.0
6 months after surgery	20	12	All follow-up patients maintained a symptom-free state	12	100.0

### 3.3 Patient satisfaction and acceptance of surgery

All 20 patients accepted endoscopic excision for internal or mixed hemorrhoids. Postoperatively, all patients expressed satisfaction with the procedure. Both satisfaction and acceptance rates were 100% (Table 5). None of the 20 patients had a recurrence, and no complications occurred.

**TABLE 5:** Patient acceptance and satisfaction following endoscopic excision.

Assessment indicators	Total cases	Number of cases with satisfaction/acceptance	Number of cases with dissatisfaction/refusal	Satisfaction/Acceptance (%)
Treatment regimen acceptance	20	20	0	100.0
Postoperative patient satisfaction	0	0	0	100.0

### 3.4 Postoperative pathological results

Postoperative pathological examinations in all 20 patients indicated anorectal masses with changes consistent with hemorrhoidal tissue. Among these patients, one patient with Grade III internal hemorrhoids had concurrent fibroepithelial polyp tissue identified. One patient with Grade IV internal hemorrhoids demonstrated pathological findings consistent with condyloma acuminatum changes. One patient with mixed hemorrhoids demonstrated viral wart tissue accompanied by submucosal vascular dilation and congestion.

## 4 Discussion

Hemorrhoids are among the most common anorectal conditions encountered in clinical practice and are characterized by high prevalence and diverse symptoms that substantially affect the quality of life of patients. Conservative treatments, including dietary modification, sitz baths, and topical medications, have limited effectiveness for moderate to severe hemorrhoids (Zhou et al., 2023). Although conventional surgical procedures can achieve complete resolution of symptoms, they also cause considerable tissue trauma, significant postoperative pain, prolonged recovery, and potential complications such as anal stenosis or fecal incontinence (Brusciano et al., 2020). With the advancement of digestive endoscopy technology, the clinical role of endoscopy in hemorrhoid management has expanded from diagnostic evaluation to therapeutic intervention.

Findings indicate that endoscopic rubber band ligation (ERBL) and endoscopic sclerotherapy are currently the most representative minimally invasive endoscopic approaches (Ma et al., 2020). ERBL provides clear visualization, accurate positioning, and strong operability, making it a suitable option for Grade II and Grade III internal hemorrhoids. However, its effectiveness decreases in patients with pronounced prolapse or a substantial component of mixed hemorrhoids, particularly when hemorrhoids are large, when ligation bands dislodge, or when tissue below the dentate line is involved, which increases the likelihood of postoperative pain and bleeding (Rao and Nashwan, 2024).

Endoscopic sclerotherapy also demonstrates favorable outcomes, particularly for Grade I and Grade II internal hemorrhoids (Gallo et al., 2022). Cap-assisted endoscopic sclerotherapy (CAES), developed by Zhang et al., incorporates a transparent cap that stabilizes the endoscopic tip and enhances visual control, thereby improving injection precision and allowing the sclerosant to act directly on the hemorrhoidal vascular plexus. This advancement has improved safety and reduced complications such as pain and infection (Wang et al., 2024). However, the short-term clinical outcomes of sclerotherapy is constrained by inadequate mucosal fixation, limited short-term clinical outcomes in treating prolapsed or mixed hemorrhoids, and recurrence in some patients within 1 year of treatment.

Given these limitations, the Department of Gastroenterology of Zhoushan Hospital explored endoscopic excision for internal and mixed hemorrhoids. This technique removes diseased submucosal tissue directly through endoscopic resection and therefore addresses the lesion more fundamentally. Compared with traditional surgical excision, endoscopic resection is performed under direct endoscopic visualization, enabling precise control of resection depth and extent while minimizing damage to the sphincter and adjacent tissues. This approach is characterized by a small wound area, minimal intraoperative bleeding, and rapid postoperative recovery.

The results of this study indicate that endoscopic excision effectively improved bleeding, prolapse, and pain while maintaining low complication rates, short hospital stays, and high patient satisfaction. Importantly, this technique also enabled the collection of complete pathological specimens, providing valuable diagnostic information for identifying potential anorectal neoplasms (Mascagni et al., 2020). This capability addresses a key limitation of ERBL and sclerotherapy, which do not allow tissue sampling.

The present study demonstrates innovation in both patient selection and procedural technique. ERBL and CAES research has primarily focused on Grade II and Grade III hemorrhoids, whereas the endoscopic excision procedure evaluated here was applicable to Grade II to Grade IV internal hemorrhoids and mixed hemorrhoids, particularly in patients with significant mucosal prolapse or recurrent disease. These findings indicate that for patients with suboptimal outcomes from conventional minimally invasive treatments, endoscopic excision may serve as an effective and safe alternative. By removing pathological tissue and reinforcing mucosal support, this method may help reduce recurrence and improve long-term outcomes.

However, several limitations must be acknowledged. This investigation was a single-center retrospective analysis with a relatively small sample size and lacked a randomized control group. Therefore, the findings require validation through large-scale multicenter prospective studies. Additionally, surgical outcomes may vary based on the technical expertise of the operator, and the reproducibility of the procedure warrants further evaluation. The relatively short follow-up period in this study also limits the evaluation of long-term recurrence and postoperative complications. Future research should include comparative analyses involving ERBL, CAES, and surgical excision, as well as standardization of operative techniques and treatment protocols to better define optimal management strategies for different hemorrhoid types.

## 5 Conclusion

Endoscopic hemorrhoidectomy demonstrated favorable outcomes in the management of Grade II to Grade IV internal hemorrhoids and mixed hemorrhoids. The procedure may offer advantages in symptom relief, reduction of recurrence, pain mitigation, and postoperative recovery, thereby broadening the scope of minimally invasive endoscopic treatment options for hemorrhoid management. It is anticipated to complement existing modalities such as ERBL and CAES in establishing a comprehensive endoscopic therapeutic system for hemorrhoids.

## Data Availability

The original contributions presented in the study are included in the article/Supplementary Material, further inquiries can be directed to the corresponding author.

## Supplementary material

SUPPLEMENTARY MATERIAL VIDEO

https://doi.org/10.6084/m9.figshare.31238824.
